# Opioid exit plans for tapering postoperative pain control in noncancer patients: a systematic review

**DOI:** 10.1186/s13037-024-00408-w

**Published:** 2024-07-30

**Authors:** Marcel Rainer, Sarah Maleika Ommerli, Andrea Michelle Burden, Leo Betschart, Dominik Stämpfli

**Affiliations:** 1 Institute of Pharmaceutical Sciences, ETH Zurich, Vladimir-Prelog Weg 1-5/10, 8093 Zurich, Switzerland; 2 Hospital Pharmacy, Department Medical Services, Kantonsspital Baden, Im Ergel, 5404 Baden, Switzerland; 3 Chemistry | Biology | Pharmacy Information Center, ETH Zurich, Vladimir-Prelog Weg 10, 8093 Zurich, Switzerland

**Keywords:** Systematic review, Opioid tapering, Deprescribing, Drug safety, Opioid analgesics, Hospital discharge, Noncancer pain, Transitional care, Postoperative patient management, Preventive medicine

## Abstract

**Background:**

A growing number of countries have reported sharp increases in the use and harm of opioid analgesics. High rates of new opioid initiation are observed in postoperative patients. In response, various tertiary care institutions have developed opioid exit plans (OEPs) to curb potential opioid-related harm.

**Methods:**

PubMed and Embase were systematically searched to identify, summarize, and compare the interventional elements of OEPs for postoperative patient populations published from January 1, 2000, to June 4, 2024. Two researchers independently screened the articles for eligibility following the PRISMA 2020 guidelines, extracted the data, and assessed the study quality and risk of bias. Data synthesis was performed for study characteristics, intervention details, efficacy, and development.

**Results:**

A total of 2,585 articles were screened, eight of which met the eligibility criteria. All studies were conducted in North America and focused on orthopedic surgery patients following total hip or knee arthroplasty (n = 5) or neurosurgery (n = 3). Most studies (n = 7) included a pre-post (n = 4) or randomized clinical design (n = 3). Three studies were of good quality, and none had a low risk of bias. The interventions varied and ranged from educational sessions (n = 1) to individualized tapering protocols (n = 4) or a combination of the two (n = 2). Key elements were instructions on how to anticipate patients’ postoperative need for opioid analgesics and tapering strategies based on 24-h predischarge opioid consumption. Six studies included efficacy as an endpoint in their analysis, of which four assessed statistical significance, with all four identifying that the OEPs were successful in reducing postoperative opioid use.

**Conclusion:**

Despite differences in design and implementation, the identified OEPs suggest that they are efficacious in reducing outpatient opioid consumption. They provide a robust estimate of postoperative analgesic requirements and a rationale for tapering duration and rate. However, more rigorous studies are needed to evaluate their real-world effectiveness.

**Supplementary Information:**

The online version contains supplementary material available at 10.1186/s13037-024-00408-w.

## Background

Over the past two decades, opioid overdoses have claimed hundreds of thousands of lives, with millions grappling with opioid use disorder [1, 2]. Analyses of drug monitoring systems have revealed high rates of new opioid prescriptions among postoperative patients and within family medicine [3–9]. While the US opioid crisis is largely fueled by illicit opioid use (i.e., fentanyl), it is a result of an ongoing epidemic rooted in high rates of prescription opioid use [2].

Europe now witnesses a similar surge in prescription opioids [10–20], resulting in an increased incidence of opioid-related harms associated with opioid overconsumption, defined as prolonged use or higher doses for noncancer pain [21–24]. Notably, prolonged use may develop rapidly among opioid-naïve users [25–27]. Despite lower rates of opioid-related deaths in Europe than in the US, early intervention is crucial to prevent a shift from prescription to illicit opioids, as health policies alone may not suffice [28, 29].

Opioid stewardship programs have emerged in North America as a response to the prescription opioid crisis, employing strategies to decrease and track opioid prescriptions [30, 31]. These have been effective in reducing the number of opioid prescriptions or tablets without compromising patient well-being [32–34]. At their core, these programs incorporate opioid exit plans (OEPs), consisting of specific strategies that promote drug safety for improved outcomes, closing an important prevention gap.

While some countries are developing guidelines for opioid analgesic deprescribing [35–38], a recent guideline summary identified a need for greater evidence on the effectiveness of current strategies to inform clinical practice [35]. Therefore, this systematic review aimed to identify and summarize published hospital-based OEPs, detailing their design, main components, and reported evidence of their effectiveness.

## Methods

A systematic review was performed according to the PRISMA 2020 guidelines [39] and the SPICE (setting, population, intervention, comparison, evolution) [40] and PCC (population, concept, context) [41] frameworks to define the study environment. The search was conducted in PubMed and Embase using a distinct keyword search string developed with an information specialist (LB). Articles published from January 1, 2000 to June 3, 2024, that explored the discharge management of postoperative patients receiving opioid analgesics were considered eligible. For homogeneous interventional exposure, articles needed to focus on patients 18 years of age or older at discharge, excluding patients with special needs or implications for routine outpatient opioid use after surgery, such as cancer, end-of-life care, and substance use disorders. The articles needed to include an accessible tapering protocol. The full search strategy and list of eligibility criteria for the literature are detailed in the Supplement Tables 1, 2, and 3.

Two searches were conducted (SO, MR), one on April 27, 2023, and an update on June 4, 2024. The results were imported into Rayyan.ai for screening [42] and duplicates were removed. Two researchers (SO, MR) independently screened the abstracts and obtained full-text articles if the predefined eligibility criteria were met. Conflicts in screening were resolved through in-person discussions. If necessary, a third author (DS) was consulted. The Cochrane Effective Practice and Organization of Care Group [43] template was used for consistent and comprehensive data collection on study characteristics and measured intervention efficacy (SO, MR), reported as a percentage reduction in opioid dosage as morphine milligram equivalents (MME) when applicable.

Three reviewers (SO, DS, MR) appraised the quality of evidence of the included studies using the LEGEND (let evidence guide every new decision) evidence evaluation tool [44]. In the LEGEND, a numerical rating system based on the study design determines the basic grading. Indicators "a" and "b" differentiate the quality of evidence: "a" indicates high quality, while "b" indicates inconsistencies or insufficient quality of design [44]. Disagreements in grading were resolved during in-person discussions. If a reported study design was suspected to be incorrect, three reviewers (SO, DS, MR) collectively reclassified the study.

When applicable, two reviewers (MR, DS) independently applied the Revised Risk of Bias tool (RoB2) for randomized controlled trials (RCTs) [45] and the Risk Of Bias In Non-randomized Studies—of Interventions (ROBINS-I) tool for non-randomized studies [46] to identify potential biases and confounders, assessing the level of risk.

## Results

### Article selection

**Fig. 1:**
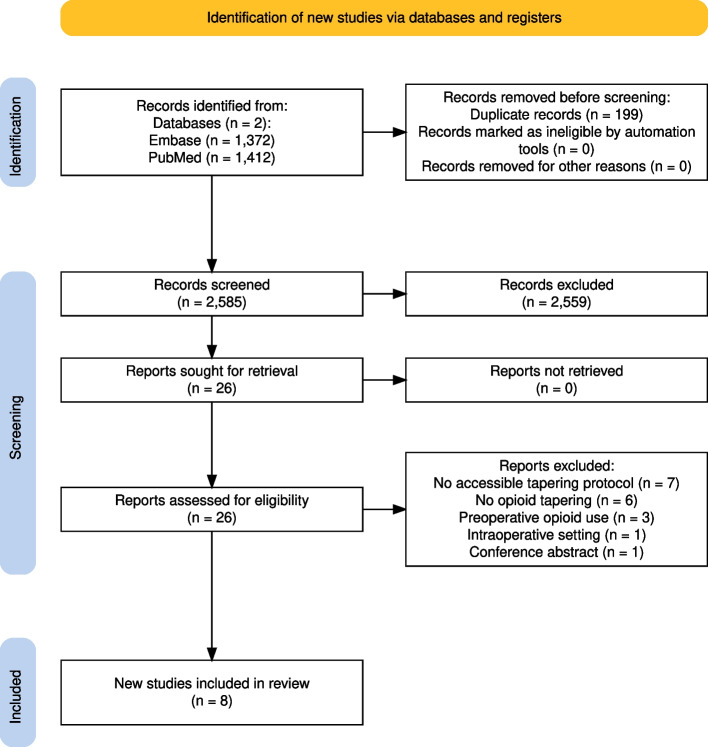
PRISMA flow diagram of screening and inclusion process [39, 47]

Figure 1 illustrates the screening and inclusion process [39, 47]. The initial systematic literature search identified 2,483 articles, and the updated search identified 102 articles (n = 2,585). The respective abstracts were screened, and 26 articles were deemed eligible for full-text screening. Eventually, eight articles from the full-text screening were included in the final analysis [48–55].

### Study characteristics

Table 1 provides an overview of the characteristics of the eight included studies. All the articles described studies conducted in North America, with 25% (N = 2) in Canada [48, 50] and 75% (N = 6) in the US [49, 51–55]. Half of the studies (N = 4) were quality improvement studies [51, 53–55] that were either uncontrolled and retrospective [53–55] or controlled and prospective [51]. Three were RCTs (37.5%; N = 3) [48–50], and one was a proposed OEP for patient services targeting postoperative pain [52]. For the latter, no conventional study design could be assigned. While the procedures varied, the studies predominantly investigated interventions within orthopedic departments, with total hip arthroplasty (THA) and total knee arthroplasty (TKA) being the most prevalent procedures (75%; N = 6) to involve patients in OEPs [48–51, 54, 55], followed by neurosurgery (12.5%; N = 1) [53]. The proposed OEP framework by Genord et al. [52] was considered applicable to orthopedic, neurosurgical, and colorectal surgery.

**Table 1:** Study characteristics of the included studies using the template provided by the Cochrane Effective Practice and Organization of Care Group [43] and quality assessment of different study types using the LEGEND (let evidence guide every new decision) evidence evaluation tool [44]. The numbers in the QA column represent the study design used, and the letters indicate that a is of good quality or b is of lesser quality

Study	Study type	Study location	Study setting	Study population	Patient demographics	Total number of patients	QA
**Bérubé et al. 2022 **[48]	Pilot-RCT	Canada	Level-1 trauma center	Patients with traumatic injury and an increased risk for chronic opioid consumption	Mean age (SD): I: 41.1 (18.5) Pamphlet: 40.2 (16.2) % male: I: 76% Pamphlet: 75%	N = 50	2b
**Hah et al. 2020 **[49]	Pilot-RCT	United States	Academic medical center	Patients undergoing TKA or THA	Mean age (SD): UC: 66.2 (8.6) I + UC: 64.8 (8.8) % male: UC: 43.6% I + UC: 53.1%	N = 104	2a
**Singh et al. 2018 **[50]	RCT	Canada	Acute care and teaching hospital	Patients with no chronic pain conditions and no opioid abuse history undergoing elective orthopedic surgery (foot and ankle surgery)	Mean age (range): Total population: 50.68 (2 to 65) No. male/total: 17/80	N = 80	2b
**Chen et al. 2020 **[51]	Quality improvement study *(prospective controlled pre-post design)*	United States	1) Urban safety-net hospital and level I trauma center 2) Suburban, community hospital and level II trauma center	Orthopedic surgery patients and nonorthopedic surgery patients	*Orthopedic patients* Mean age (range): Pre: 55.9 (0 to 92) Post: 57.1 (6 to 88) % male: Pre: 46% Post: 48% *Nonorthopedic patients:* Mean age (range): Pre: 54.4 (0 to 100) Post: 54.1 (0 to 99) % male: Pre: 47% Post: 48%	N = 22,083	4a
**Genord et al. 2017 **[52]	N/A *(Summary- and example report on the implementation of a pharmacist-led OEP)*	United States	Hospital	Orthopedic surgery patients (TKA, THA), neurosurgery patients (fusion, 2 levels or greater), colorectal surgery patients (all)	N/A	N/A	5b
**Joo et al. 2020 **[53]	*Reclassified:* Quality improvement study *(retrospective uncontrolled pre-post design)*	United States	Tertiary care university-affiliated VA hospital	Spine surgery patients	Median age (IQR): Pre: 67 (61–70) Post: 66 (61–72) % male: Pre: 99% Post: 100%	N = 83	4b
**Kukushliev et al. 2022 **[54]	Quality improvement study *(retrospective uncontrolled pre-post design)*	United States	Tertiary care university-affiliated VA hospital	Patients undergoing TKA or THA	Mean age (SD): Pre: 67 (8.3) Post: 67 (9.6) % male: Pre: 93.6% Post: 96.6%	N = 388	4b
**Tamboli et al. 2020 **[55]	*Reclassified:* Quality improvement study *(retrospective uncontrolled pre-post design)*	United States	Tertiary care university-affiliated VA hospital	Patients undergoing THA	Median (10th–90th percentiles) Pre: 67 (58–72) Post: 69 (56–73) % male: Pre: 96% Post: 92%	N = 49	4a

*N/A* Not applicable, *N* Sample size, *OEP* Opioid exit plan, *TKA* Total knee arthroplasty, *THA* Total hip arthroplasty, *PDMP* Prescription drug monitoring program, *MI* Motivational interviewing, *VA* Veterans affairs, *QA* Quality assessment: 2 = randomized clinical trial, 4 = longitudinal study, 5 = published expert opinion; a = good quality, b = lesser quality. Pre: Preintervention group. *Post* Postintervention group, *RCT* Randomized clinical trial, *SD* Standard deviation, *UC* Usual care group, *I* Intervention group, *IQR* Interquartile range

The patient demographics varied largely within the study populations and the reported items due to differences in study design (Table 1). Across the studies, patients had a mean age between the mid-fifties and mid-sixties, with the lowest mean age being 40.2 years [48] and the highest being 67.0 years [53–55]. The gender distribution was rather balanced in three studies [48, 49, 51], whereas studies conducted in Veterans Affairs Facilities [53–55] predominantly included male patients, and the study by Singh et al. [50] predominantly included female patients. A history of substance abuse, financial stability, mood disorders, preoperative pain, or prior opioid use was reported by 75% of the studies [48, 49, 51, 53–55]. Most studies reported psychiatric comorbidities (62.5%; N = 5) [48, 49, 53–55]. This was either done by screening for anxiety and depressive disorders (25%; N = 2) [48, 49], or the screening and the exact entity were not specified [53–55]. Kukushliev et al. [54] were the only ones to report further comorbidities, such as cardiovascular, renal, or hepatic diseases or impairments.

### Quality of the included studies

Table 1 reports the quality of evidence for each study. Three [49, 51, 55] studies were found to be of good quality. The studies by Hah et al. [49], Chen et al. [51], and Tamboli et al. [55] selected an appropriate study method for the research question. These reported statistically significant results while also describing the intervention, patient allocation, variables, and outcomes clearly. The remainder received lower quality ratings, mostly due to underreporting of important details such as intervention delivery and the randomization process.

### Risk of bias

**Fig. 2:**
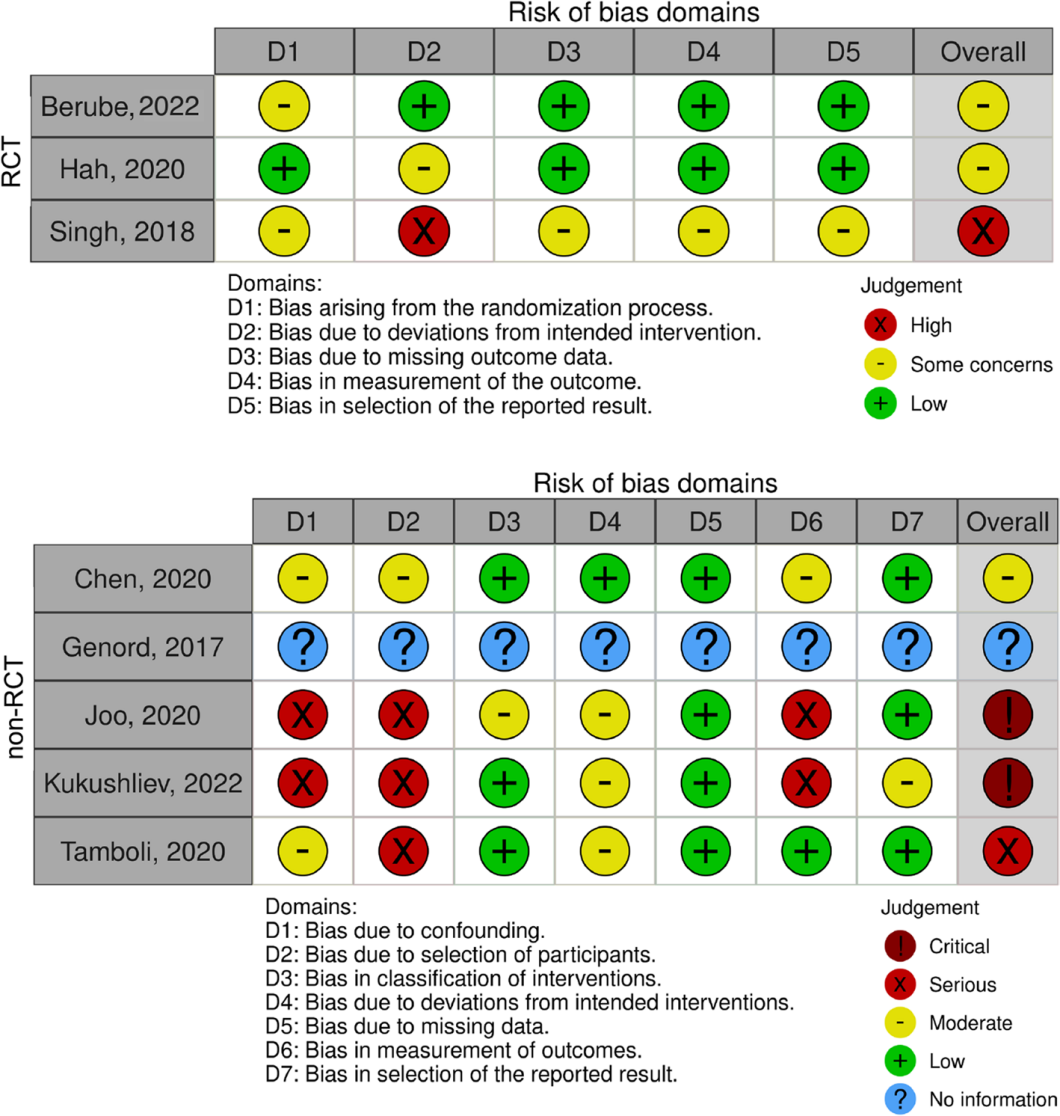
Visualization of the risk of bias assessments in the respective domains (D) [56] using the Revised Risk of Bias tool [45] for randomized controlled trials (RCTs) and the Risk Of Bias In Non-randomized Studies—of Interventions tool [46] for non-randomized studies (non-RCTs)

Figure 2 [56] visualizes the bias judgments. All studies had a moderate to high risk of bias. The RCT by Hah et al. [49] was the only RCT with good quality evidence and moderate bias. However, there were some concerns regarding deviations from the intended protocol intervention. Among the non-RCT studies, the studies by Chen et al. [51] and Tamboli et al. [55] were both high quality. However, there were moderate to serious concerns regarding confounding, participant selection, outcome measurement, and protocol deviations.

### Overview of interventions and outcome assessment

Table 2 provides the details of the intervention strategies. The most common (75%, N = 6) feature was an individualized tapering approach [50–55]. Tamboli et al., Joo et al., and Kukushliev et al. [53–55] used patients’ 24-h predischarge opioid utilization to generate a patient-specific tapering plan. In the pre-post design study by Chen et al. [51], the intervention was a model that converted 24-h predischarge opioid utilization to the preferred opioid analgesic for discharge and to the preferred tapering duration in days (0, 7, or 14 days) depending on the type of surgery. Singh et al. [50] assigned patients to risk groups for postoperative pain with risk group-specific tapers based on procedure type, which focused on postoperative patient satisfaction rather than on reducing the amount of opioids prescribed at discharge. Contrary to individualizing tapering regimens, Hah et al. [49] employed postoperative motivational interviewing to promote patients’ efforts toward medication adherence, opioid tapering, and pain management while closely monitoring pain outcomes and opioid-related adverse events.

**Table 2:** Summary of the interventions and details of the outcome assessments among the included articles using the template provided by the Cochrane Effective Practice and Organization of Care Group [43]

Study	Intervention components and timing	Opioid type	Taper speed	No. of tapering days	Intervention description	Comparator	Intervention efficacy
**Bérubé et al. 2022 **[48]	- Two educational sessions within the week before hospital discharge - A maximum of six opioid tapering counseling sessions every two weeks following discharge	NR	25% dose reduction per day until opioid cessation	N/A	**Aim:** To prevent and reduce chronic opioid use in high-risk trauma patients **Intervention:** Two 10-min educational sessions within the week before hospital discharge, and a maximum of six 15-min opioid tapering counseling sessions every two weeks following discharge	Standard pain management and an educational pamphlet	**Primary outcome on feasibility:** Median score for all acceptability dimensions = 3 (0–4) **Secondary outcomes on efficacy:** Patient-reported opioid use at 6 weeks reduced to 1.2% from baseline (MED 106.8 (SD 46.9) and at 12 weeks reduced to 0.4% from baseline MED 106.2 (SD 86.0). The comparison group reduced opioid use to 12% and later to 4%
**Hah et al. 2020 **[49]	**Timing:** Starting at 14 days postsurgery, the intervention is delivered once per week until week seven. If opioid cessation has not been reached by this time, the intervention continued with a monthly frequency up to one year **Components:** - Motivational interviewing - Guided opioid tapering support	NR	- Dose reduction of 25% of the total opioid dose every seven days starting 14 days after surgery - If pain worsened (NRS > 7), the dose was increased by 25% for seven days, and a reassessment was made - If there were signs of withdrawal (SOWS mean score > 2), the dose was held for seven days, and a reassessment was made - Upon reaching one opioid tablet per day, opioid cessation was to be achieved within the next seven days	N/A	**Aim:** To accelerate the return to preoperative baseline opioid doses and to achieve definitive opioid cessation faster **Intervention:** MI and guided opioid tapering support via telephone MI part: reviewing medication adherence prior to the phone call, reviewing response to medication, giving advice concerning opioid weaning, providing support for patient’s efforts, educating on pain management and drug misuse, and discussing nonadherence Guided opioid tapering support: guiding and monitoring opioid weaning after hospital discharge	Usual care, a review session of the standard medication instructions from 14 days after surgery, and identical follow-ups	**Primary outcome on return to baseline MME:** Mean time to baseline opioid use reduced by 30 days (67.8 days UC group, 34.6 days intervention group) Rate of return to baseline opioid 62% higher in the intervention group (HR 1.62; 95% CI 1.06–2.46; *p* = 0.03) Intervention resulted in a 53% increase in the rate of complete postoperative opioid cessation (HR 1.57; 95% CI 1.01–2.44; *p* = 0.05)
**Singh et al. 2018 **[50]	Pamphlet with written instructions for the management of postoperative pain and an opioid tapering schedule, both provided at hospital discharge	Hydromorphone, oxycodone/acetaminophen or tramadol/acetaminophen	NR	7, 14, or 21 depending on the risk group	**Aim:** To augment the effect of the already established ERAS protocol at the institution and to reduce the amount of opioids used by patients after surgery postdischarge **Intervention:** A patient-specific protocol instructing the patient on how to taper their postoperative opioids based on the amount of opioid consumed during the 24-h period prior to discharge	Discharge prescriptions for 30, 60, or 90 tablets of oxycodone 5 mg or hydrocodone-acetaminophen 5 mg to be taken every 4–6-h on an as-needed basis	NR
**Chen et al. 2020 **[51]	- Taper calculator for prescribers that generates a patient-specific taper based on the patient’s 24-h predischarge opioid utilization - Patient-specific tapering plan provided at hospital discharge	Oxycodone (but method can be used for various opioids)	Use of a taper calculator to reduce the daily maximum dose according to the formula below to a daily maximum down to 10% of the patient’s 24-h predischarge opioid dose at the completion of the taper $y=A*{e}^{\frac{-\text{ln}10}{L}*t}$ *y: daily maximum limit* *A: 24-h predischarge opioid utilization (in oral MME)* *L: total length of the taper (in days)* *t: postdischarge day*	7 or 14 days depending on the type of surgery	**Aim:** To standardize opioid prescribing at discharge after inpatient orthopedic surgery and to reduce the quantity of opioids prescribed at discharge **Intervention:** An opioid taper calculator generating a personalized opioid taper for patients based on their 24-h predischarge opioid utilization	NR	**Primary outcome on discharge quantity:** 427 MME pre to 326 MME post implementation (*p* < 0.001), 24% reduction **Same outcome for nonorthopedic patients:** 252 MME pre to 229 MME post (*p* = 0.032), 9% reduction
**Genord et al. 2017 **[52]	Before surgery: - PDMP search - Preoperative medicine reconciliation review Inpatient period: - Inpatient postoperative treatment plans - Discharge treatment planning At/after discharge: - Discharge counseling - Follow-up appointments	Oxycodone (but method can be used for various opioids)	No predefined taper rate or any specific equation Approximate overview: Home days 1–4: Same dose per day as the 24-h predischarge dose (starting dose) Home days 5–7: Approximately a 20–30% dose reduction Home days 8–10: Doses were approximately at approximately 50% of the starting dose, except for very low starting doses which were approximately 75% of the starting dose	10 days	**Aim:** To evaluate the impact of written discharge instructions on patient pain satisfaction, minimizing opioid risk exposure, number of patients seeking a renewal prescription, and on appropriate disposal of leftover prescription medication in orthopedic surgery **Intervention:** Written discharge instructions for postoperative pain management in form of a pamphlet which summarized postoperative pain expectations and provided recommendations for opioid medication indication, usage, and disposal of left-over tablets	Same discharge opioid prescriptions (= the same number of tablets) corresponding to a previously assigned risk group, but no written discharge instructions	NR
**Joo et al. 2020 **[53]	Patient-specific tapering plan provided at hospital discharge	Described for oxycodone	Home days 1–2: Same dose per day as the 24-h predischarge dose (starting dose) Then: The daily dose was reduced by 10 mg every second day until opioid cessation	12 days	**Aim:** To improve management of acute postoperative pain during all phases of hospital stay and at discharge **Intervention:** PDMP search, preoperative medicine reconciliation review, inpatient postoperative treatment plans, discharge treatment planning (including personalized education material and tapering instructions for patients), discharge counseling, and follow-up appointments	N/A	**Primary outcome on opioid dosage:** Median MME through six weeks after surgery was 630 pre to 280 post implementation (*p* < 0.01) **Secondary outcome on discharge quantity:** 900 MME pre to 300 MME post implementation (*p* < 0.01), 66% reduction
**Kukushliev et al. 2022 **[54]	Personalized tapering protocol provided at hospital discharge	Described for oxycodone	Home days 1–2: Same dose per day as the 24-h predischarge dose (starting dose) Then: The daily dose was reduced by 10 mg every second day until opioid cessation	12 days	**Aim:** To decrease the total dose of opioids prescribed postdischarge after elective primary spine surgery **Intervention:** A patient-specific discharge opioid prescribing and tapering protocol based on the patient’s 24-h prior to discharge oral opioid consumption	NR	**Primary outcome on discharge quantity:** Overall 224 MME pre to post reduction (for non-opioid-naïve 366 ME and opioid-naïve 209 MME) Greater reduction in TKA patients (266 MME) than in THA patients (136 MME)
**Tamboli et al. 2020 **[55]	Patient-specific tapering plan provided at hospital discharge	Described for oxycodone	Home days 1–2: Same dose per day as the 24-h predischarge dose (starting dose) Then: The daily dose was reduced by 10 mg every second day until opioid cessation	12 days	**Aim:** To decrease the total dose of opioids prescribed postdischarge after THA **Intervention:** A patient-specific discharge opioid prescribing and tapering protocol based on the patient’s 24-h prior to discharge oral opioid consumption	NR	**Primary outcome on opioid dosage:** Median MME through six weeks after surgery was 900 pre to 295 post implementation (*p* = 0.007) Mean difference of 721 MME (reduction of 63% pre vs post) **Secondary outcome on discharge quantity:** 675 MME pre to 180 MME post implementation (*p* = 0.003) Mean difference (95% CI) of 387 MME pre vs post

*CI* Confidence interval, *ERAS* Enhanced recovery after surgery, *MED* Morphine equivalent dose, *MI* Motivational interviewing, *MME* Morphine milligram equivalents, *N/A* Not applicable, *NR* Not reported, *NRS* Numeric rating scale, *OEP* Opioid exit plan, *PDMP* Prescription drug monitoring program, *Pre* Preintervention group, *Post* Postintervention group, *SD* Standard deviation

The articles by Bérubé et al. [48, 57] and Genord et al. [52] describe combined interventions that extended beyond primarily comprising a tapering protocol (Table 2). Bérubé et al. [48, 57] emphasized educational interventions. Patients participated in face-to-face educational sessions prior to discharge and thereafter, focusing on multimodal pain management and guidance on opioid tapering. Pain levels and interference with daily life were closely assessed after hospital discharge and complemented with generic tapering recommendations. These efforts aimed to improve patients’ self-management. At discharge, patients received an educational pamphlet with the aforementioned information. Genord et al. [52] proposed a yet to be trialed three-phase OEP to support opioid cessation. The first phase, prior to discharge, will include interdisciplinary rounds to assess analgesic needs and discharge eligibility. In the second phase, patients receive discharge counseling and an individualized pain management plan. In the third and final phase after discharge, patients will undergo medication evaluations based on progress with the prescribed pain regimen, opioid discontinuation status, and opioid-related adverse events.

All the published OEPs were developed for standard opioid analgesics (Table 2) using various decreasing approaches. Most studies did not restrict inclusion based on opioid type. Chen et al. [51] provided opioid conversion factors to taper the preferred opioid, and Singh et al. [50] included a predefined set of opioids (hydromorphone, oxycodone/acetaminophen, tramadol/acetaminophen). Hah et al. [49] and Bérubé et al. [48] did not specify. Genord et al. [52] proposed an untrialed tapering regimen to be applicable to any opioid analgesic. The studies based on the tapering regimen by Tamboli et al. [53–55] (Table 2) focused specifically on oxycodone. The OEP regimens followed either a linear [50, 53–55], exponential [48, 49, 52], or logarithmic [51] reducing tapering approach. The duration was either fixed for the investigated patient population [52–55] or adapted to the type of procedure [50, 51], while Hah et al. [49] and Bérubé et al. [48] did not predetermine a day of opioid or tapering cessation.

Table 2 also provides an overview of the primary endpoints. Overall, six of the eight studies assessed the efficacy of OEPs on opioid reduction or pain [48, 49, 51, 53–55], of which four reported statistical significance [49, 51, 53, 55]. Tamboli et al., Joo et al., and Kukushliev et al. [53–55] demonstrated a decrease in the dosage of opioids as MME of 56% (630 vs 280 MME, *p* < 0.01) and 63% (900 vs 295 MME, *p* < 0.01) within six weeks of postoperative discharge in the preintervention period and postintervention period, respectively. Similarly, compared to the preintervention period, the approach by Chen et al. [51] resulted in a 24% reduction in the quantity of opioids consumed at discharge (427 vs. 326 MMEs, *p* < 0.001). After discharge, the authors reported the rate of opioid refills within 30 days (1.58 vs 1.71 mean number, *p* = 0.082) rather than reductions in MME. An RCT by Hah et al. [49] found that patients receiving motivational interviewing and opioid taper support were 62% more likely to return to baseline opioid use than patients in the standard care group (hazard ratio 1.62, 95% confidence interval 1.06–2.44). Detailed information on the intervention content and provider delivery is provided in Supplement Table 4.

## Discussion

This systematic review identified and summarized eight published OEPs [48–55] from hospital settings, providing concepts for the development of novel OEPs in tertiary care settings. Despite the heterogeneity of the approaches investigated, all articles that reported hypothesis testing of their primary outcomes [48–51, 53–55] were successful in achieving either a reduction in opioids at or after discharge. While none of the studies had a low risk of bias, three were of high quality according to the LEGEND quality assessment tool. All good-quality studies [49, 51, 55] yielded statistically significant results, demonstrating that the use of OEPs could effectively reduce the quantity of opioids used at or after discharge. This review therefore highlights that the application of OEPs in clinical practice could be an important addition to reducing discharge opioid consumption.

In this review, no standard OEP approach was identified, as individualization of the intervention and tapering appeared to be integral to meeting a patient’s individual analgesic need during deprescribing. This finding is in line with current evidence-based guidelines [35, 58, 59], as factors such as preoperative opioid use, preexisting pain conditions, social status, psychological comorbidities, and procedure types greatly influence pain and the risk of prolonged opioid use [60–63]. Among the identified OEPs in this review, implemented strategies included procedure-specific risk groups [50], total 24-h predischarge opioid consumption [51–55], or common pain and withdrawal assessments combined with taper counseling [48, 49]. Using 24-h predischarge opioid consumption is the most common approach and is a time-saving and practical way to individualize tapering, as the need for analgesia typically decreases as patients recover from surgery. This method has limitations, notably, its inapplicability to patients with a shorter inpatient stay than 24 h. Additionally, a shorter postoperative stay can affect pain assessments, as the residual effects of anesthesia may not have fully dissipated [63, 64]. In contrast, Hah et al. [49] and Bérubé et al. [48] employed standardized tapering rates but still individualized the tapering by continuous and close patient contact through follow-ups. The repeated assessment of pain and withdrawal symptoms during follow-up sessions facilitated adjusting the tapering to the patients’ needs. As a result, this method appears to be suitable even for complex cases and ensures sustained positive patient outcomes. Finally, Hah et al. [49] halved the time to baseline opioid use, reflecting the success of such an approach. This approach is also promoted in the American Center for Disease Control guidelines, suggesting that patients with acute pain who receive opioids for a longer time should be evaluated with a two-week frequency [59].

Although, Singh et al. [50] did not assess the statistical significance of their intervention, the OEP included an interesting element of risk stratification in opioid tapering. They allocated patients to one of three risk groups according to procedure type and anticipated postoperative opioid use to prescribe the total number of opioid tablets. A large meta-analysis including 37 studies with 1,969,953 surgery and trauma patients showed that patient-specific opioid requirements were the risk factors with the strongest association with developing chronic opioid use [62]. The American Centers for Disease Control proposed a 6- to 15-day opioid prescription for musculoskeletal procedures [65]. While stratification by procedure type may facilitate the estimation of the ideal number of opioid tablets to be prescribed at discharge, it does not address individual analgesic needs such as patient-specific opioid requirements, which are captured by reviewing 24-h opioid use prior to discharge. It may be promising to combine elements of stratification according to procedure and risk by creating risk groups based on key risk factors for chronic pain and prolonged opioid use. Opioid quantities can be minimized using 24-h inpatient opioid consumption and further individualized by dividing patients into different risk groups: if two patients have the same 24-h inpatient opioid use but one patient is in a higher risk group, the higher risk patient would have a slower tapering rate and more intensive follow-up.

Notably, this review focused on the application of OEPs in postoperative patients. In addition to chronic primary pain, noncancer postoperative patients are subject to the introduction of prescription opioid analgesics or to a higher dose than before admission [4–9]. Karmali et al. [66] showed that postoperative pain management is a key driver of long-term opioid use. Relevant predictors [60, 66, 67] for long-term opioid therapy, such as history of substance abuse, financial stability, mood disorders, preoperative pain, or preoperative opioid usage, were reported in almost all studies (75%; N = 6) [48, 49, 51, 53–55]. The study designs showed efficacy for surgical specialties associated with high invasiveness, such as orthopedic and spine surgery. For example, in orthopedic surgery, recommendations for the number of tablets range from 0 to 40 tablets of 5 mg oxycodone [68, 69]. This is equivalent to 0 to 300 MME. The described studies that measured the efficacy and the MME [51, 53–55] were approximately within the recommended postdischarge dose after the implementation of the tapering interventions. This suggests that tapering protocols have a positive influence on prescribing behavior toward guideline-recommended doses and that psychosocial aspects should be assessed. Thus, OEPs should be considered for implementation in “Enhanced Surgical Recovery” protocols as a valuable addition to patient safety, similar to opioid-free anesthesia [70]. These efforts may have a synergistic effect on opioid-sparing, as these have demonstrated in RCTs to reduce the requirement of postoperative analgesia [71–73].

There was a lack of high-quality studies, and none of the included OEPs were deemed to have a low risk of bias. Most articles lacked detailed information on the process, the rationale behind developing the tapering interventions, and consistent reporting of study endpoints. Bias concerns in RCTs mainly stemmed from randomization and intervention adherence. Some studies had predictable allocation [48] or lacked sequence information [50], while others poorly documented deviations from interventions [49, 50]. Adherence to tapering protocols was measured in only one non-RCT study [51]. It is inconclusive whether the steep logarithmic tapering method developed by Chen et al. [51] is superior to the slower linear tapering method developed by Tamboli et al. [55], or vice versa, in reducing opioid dose and improving rehabilitation outcomes. Future trials need to address these limitations and enhance the quality of the data by blinding outcome assessors. Further studies with a more rigorous study design are needed to validate the effectiveness of OEPs. The identified articles focused on the efficacy of their novel tools for assessing opioid-related outcomes, such as the number of opioid tablets taken, rather than on rehospitalization, or on extending the findings to a wider population.

The strengths of this review include the use of a robust keyword search string to screen two major medical publication platforms (PubMed and Embase). All identified articles were evaluated for quality of design and risk of bias to assess the validity of the findings. Ultimately, these findings help to reliably inform clinical practice and provide resources for the development of OEPs, allowing institutions to tailor tapering approaches to meet the needs of their patients. Limitations include the omission of articles published before 2000 and those not indexed in PubMed and Embase, including gray literature such as internal hospital guidelines and predischarge opioid-sparing protocols (e.g., enhanced recovery programs). Articles written in languages other than English or German were also excluded, as were those with inaccessible tapering protocols. Due to the eligibility criteria, the findings have limited applicability to patients with chronic opioid use and psychiatric disorders and no evidence for use in pediatrics.

## Conclusions

Despite differences in the patient populations, the studies that evaluated efficacy found that the use of OEPs with tapering plans consistently reduced opioid consumption. The 24-h predischarge method provides a robust estimate of outpatient analgesic requirements, which can be complemented by risk group stratification for tapering speed. More rigorous studies are needed to assess the effectiveness of these tapering approaches on a larger scale.

### Supplementary Information

Supplementary Material 1.

Supplementary Material 2.

## Data Availability

No datasets were generated or analysed during the current study.

## Abbreviations

LEGEND: Let Evidence Guide Every New Decision
MME: Morphine milligram equivalents
OEP: Opioid Exit Plan
PCC: Population, Concept, Context
RCT: Randomized Clinical Trial
RoB2: Revised Risk of Bias tool
ROBINS-I: Risk of Bias In Non-randomized Studies—of Interventions
SPICE: Setting, Population, Intervention, Comparison, Evolution
THA: Total Hip Arthroplasty
TKA: Total Knee Arthroplasty
