# Supplementary material for: Opioid exit plans for tapering postoperative pain control in noncancer patients: a systematic review
Source: Patient Saf Surg. 2024 Jul 30;18:25. doi: 10.1186/s13037-024-00408-w (PMC11290124; doi:10.1186/s13037-024-00408-w)
Supplement: Supplementary file 2 — Supplementary Material 2. [file 13037_2024_408_MOESM2_ESM.docx]

**Supplement**

**Opioid exit plans for tapering postoperative pain control in noncancer patients: a systematic review**

Marcel Rainer^a, b^, Sarah Maleika Ommerli*^a^, Andrea Michelle Burden^a^, Leo Betschart^c^, Dominik Stämpfli^a, b^

^a^ Institute of Pharmaceutical Sciences, ETH Zurich, Vladimir-Prelog Weg 1-5/10, 8093 Zurich, Switzerland

^b^ Hospital Pharmacy, Department Medical Services, Kantonsspital Baden, Im Ergel, 5404 Baden, Switzerland

^c^ Chemistry | Biology | Pharmacy Information Center, ETH Zurich, Vladimir-Prelog Weg 10, 8093 Zurich, Switzerland

* Contributed equally as co-first authors.

Corresponding author: Dr. Dominik Stämpfli, [dominik.staempfli@pharma.ethz.ch](mailto:dominik.staempfli@pharma.ethz.ch),

Institute of Pharmaceutical Sciences, ETH Zurich, Vladimir-Prelog Weg 1-5/10, 8093 Zurich, Switzerland

Search Strategy and Eligibility Criteria

**Table 1** Question framework to formulate a search strategy

| **Question Framework** | | |
| --- | --- | --- |
| **S/C** | **Setting/Context** | - Hospital discharge - Post-surgery period |
| **P** | **Population** | - Adult (18+), all genders - Patients with acute postoperative pain - Prescription for oral opioids at hospital discharge - Codeine, hydrocodone, dihydrocodeine, morphine, hydromorphone, oxycodone, oxymorphone, levorphanol, fentanyl, meperidine/pethidine, buprenorphine, pentazocine; tramadol, tapentadol |
| **I/C** | **Intervention/Concept** | - Opioid exit plan |
| **C** | **Comparison** | - No comparison - Intervention vs. no intervention (intervention vs. standard of care) |
| **(E)** | **(Evaluation)** | - Not part of the research question - Applies only if effectiveness was tested |
| *PCC = Population, Concept, Context*  *SPICE = Setting, Population, Intervention, Comparison, Evaluation* | | |

| ***SPICE/PCC* question framework category** | | | **Database** | **Search string code**  *(MeSH terms for Pubmed, Emtree terms for Embase)* | |
| --- | --- | --- | --- | --- | --- |
| **S/C** | **Setting/Context** | | **Embase** | 'hospital discharge'/syn OR 'surgery'/syn | |
|  |  |  | **PubMed** | "surgery"[All Fields] OR “hospital discharge”[All Fields] | |
| **P** | **Population – Subcategory “Medical Condition”** | **Population – Subcategory “Treatment”** | **Embase** | ('postoperative' OR 'post-operative' OR 'post-surgery' OR 'postsurgery' OR 'post-surgical' OR 'postsurgical') AND 'pain'/syn | 'narcotic analgesic agent'/de OR 'opiate'/de OR 'dihydrocodeine'/de OR 'codeine'/de OR 'hydromorphone'/de OR 'morphine'/de OR 'oxycodone'/de OR 'tapentadol'/de OR 'tramadol'/de OR 'fentanyl'/de OR 'buprenorphine'/de OR 'hydrocodone'/de OR 'oxymorphone'/de OR 'levorphanol'/de OR 'pentazocine'/de OR 'pethidine'/de |
|  |  |  | **PubMed** | "Pain, Postoperative/drug therapy"[MeSH] OR "Pain, Postoperative/prevention and control"[MeSH] OR (“pain”[All Fields] AND ("postoperative"[All Fields] OR "post-operative"[All Fields] OR "post-surgery"[All Fields] OR "postsurgery"[All Fields] OR "post-surgical"[All Fields] OR "postsurgical"[All Fields])) | "Analgesics, Opioid"[MeSH] OR  "Codeine"[MeSH] OR "Hydromorphone"[MeSH] OR "Morphine"[MeSH] OR "Tapentadol"[MeSH] OR "Tramadol"[MeSH] OR "Fentanyl"[MeSH] OR "Buprenorphine"[MeSH] OR "Oxymorphone"[MeSH] OR "Levorphanol"[MeSH] OR "Pentazocine"[MeSH] OR "Meperidine"[MeSH] |
| **I/C** | **Intervention/Concept** | | **Embase** | 'exit plan' OR 'taper*' OR 'wean*' OR 'drug dose reduction'/de OR 'patient counsel*'/de OR 'patient education'/de OR 'deprescription'/de OR 'opioid stewardship'/de OR (‘prescription drug misuse’ AND ‘prevention’) OR ‘instruction’ | |
|  |  |  | **PubMed** | “exit plan”[All Fields] OR “taper*”[All Fields] OR “wean*”[All Fields] OR "Drug Tapering"[MeSH] OR "Deprescriptions"[MeSH] OR "Patient Education as Topic"[MeSH:NoExp] OR “patient counsel*”[All Fields] OR “opioid stewardship”[All Fields] OR "Prescription Drug Misuse/prevention and control"[MeSH:NoExp] OR "Pain Management"[MeSH] OR “instruction”[All Fields] | |
| **(C)** | **(Comparison)** | | *The* ***“Comparison”*** *is not part of the search code as not all studies on the topic mention a comparison. This is because the comparison is often simply “standard of care” and thus not explicitly declared since it may be viewed as “no comparison”. The aim is to find all studies on the topic irrespective of whether they define this category.* | | |
| ***(E)*** | ***(Evaluation)*** | | *The* ***“Evaluation”*** *is not part of the search code as it is only an optional add-on. The aim is to find all studies on the topic irrespective of whether they include an evaluation.* | | |

**Table 2** Framework for keyword string development

| **Table 3** | **Inclusion criteria** | **Exclusion criteria** |
| --- | --- | --- |
| **Study characteristics** | | |
| Study design | All types | - |
| Type of publication | All else | - Conference abstracts - Editorials - Letters - Opinions |
| Geographic location of study | - | - |
| Language | English or German | - |
| Peer-review status | - Published - Peer-reviewed | - Gray literature - Retracted - Pendant/preprint |
| Date of publication | January 1^st^ 2000 to April 26^th^ 2023 | December 31^st^ 1999 and earlier |
| **Setting/Context** | | |
| **Setting:** Characteristics | - Hospital | - No psychiatric clinics/sanatoria - Ambulatory clinics |
| **Setting:** Hospital departments | - | - |
| **Context:** Characteristics | - Post-surgery period - Hospital discharge/hospital-to-home-transition | - Preoperative period - Intra-/perioperative period |
| **Context:** Surgery types | All other types of surgery | Obstetric |
| **Context:** Surgical technology/approach | All | - |
| **Population** | | |
| Age | Adult (≥ 18 years of age) | Studies/interventions that were **exclusively** designed for, **exclusively** tested on, or **exclusively** used on pediatric patients. |
| Gender | All genders | - |
| Ethnicity | - | - |
| Comorbidities | - | Studies/interventions that were **exclusively** designed for, **exclusively** tested on, or **exclusively** used on one of the following patient groups:   - Patients with opioid use disorder - Cancer patients - Renal dialysis patients - Chronic pain patients - Patients with psychiatric disorder |
| Medical condition | Acute postoperative pain | End of life care |
| Previous medication/medication history | - | - Previous opioid substitution therapies - Preoperative opioid use/chronic opioid use (If the intervention is **exclusively** designed for, **exclusively** tested on, or **exclusively** used for this patient group) |
| Current medication | - **Opioids:** Codeine, hydrocodone, dihydrocodeine, morphine, hydromorphone, oxycodone, oxymorphone, levorphanol, fentanyl, meperidine/pethidine, buprenorphine, pentazocine, tramadol, tapentadol - (Combination preparations) | - |
| **Intervention/Concept** | | |
| Design | - Single intervention (just the OEP) or also included if part of a collective intervention (ERAS for example) - Must include the tapering of an opioid | - |
| Form of delivery | All forms (written, digital, half-digital, print-out, telephone service, text messages, online platform, app etc.) | - |
| Development | Health care professionals | - |
| Accessibility | Must include an accessible tapering protocol. | - |
| **Comparison** | | |
| Comparator | Standard of care or no intervention | - |
| **Evaluation** | | |
| - | - | - |

**Table 4** Description of intervention delivery and the corresponding providers

| Study | Content details of intervention components | Providers | Provider experience / training |
| --- | --- | --- | --- |
| Bérubé et al.  2022 | **Educational sessions:** Information on the components of pain, how to assess pain intensity, adequate use of the prescribed analgesics, the necessity of opioid tapering to prevent abuse and dependence, cryotherapy, deep breathing relaxation exercises, staying active with the SMART procedure, and an adequate sleep hygiene.  **Counselling sessions:** Assessment of pain intensity at rest and upon movement in the last 48 h, analgesics taken over the last 72 h, non-pharmacological pain management strategies used over the last 72 h, and discussing the importance of non-pharmacological pain management strategies, follow-up on patient’s activity, instructions on how to gradually reduce opioids (if pain < 4/10 and if pain did not interfere with activities.), advice on further steps if still taking opioids after six sessions. | Trauma case manager nurse,  nurse practitioner student, or the trauma service pharmacist | **Experience:** More than five years of experience with trauma patients  **Training:** 4-hour training session by the principal investigator on the program’s details |
| Hah et al.  2020 | **Motivational interviewing:** Review of medication adherence, review of response to medication, advice concerning opioid weaning, support for patient’s efforts, education on pain management and drug misuse, and discussion of non-adherence.  **Guided opioid tapering support:** Monitoring for adverse effects of tapering including worsening pain (measured with NRS) and opioid withdrawal (measured with SOWS). If worsening pain or symptoms of withdrawal were present, the tapering plan was adjusted accordingly. | Pain medicine physician | **Experience:** NR  **Training:** The anesthesiologist received MI-training through workshops and coaching sessions every six months. |
| Singh et al.  2018 | **General instructions:** Patients are instructed to first try to control pain with Tylenol (acetaminophen) and Advil (ibuprofen), to only use opioid medication if necessary, and to dispose of left-over medications (to return extra pills to a local pharmacy).  All risk groups were instructed to use: 1) Tylenol/acetaminophen 650 mg every 6-8 hours for 3 days, 2) Advil/ibuprofen 400 mg every 8 hours for 3 days, and 3) to continue Advil and Tylenol after 3 days as needed.  **Tapering Schedule:** The opioid tapering schedule for opioid medication for each risk group included the total daily opioid tablets and the frequency for each tapering day. | NR | NR |
| Chen et al.  2020 | **Taper calculator:** The taper calculator is an Excel sheet. It includes instructions on its use for prescribers. The prescriber can enter the amount of short-acting opioids administered to the patient 24-hour before discharge, select the opioid of choice and the tablet dose, and choose the number of taper days. Based on the prescriber’s entry, the taper calculator recommends the total number of tablets to be prescribed and the number of tablets that should be used on each day of the taper. This can then be copied and pasted into the patient’s discharge instructions.  **Tapering plan:** It includes the taper day and the corresponding maximal number of 5 mg oxycodone tablets that the patient may take. It features some additional instructions (i.e. not to exceed the daily maximum etc.). It allows the patient to track the amount of opioids taken per day. | - Rotating team of orthopedic junior residents, nurse practitioners, and physician assistants *(Opioid prescriptions at inpatient discharge)*  - Nurses *(Reviewing opioid taper with patients at discharge)* | **Experience:** NR  **Training:** The orthopedic team was educated in the taper protocol. Additionally, the nurses were trained to review the taper protocol with the patients. |
| Genord et al.  2017 | **PDMP search:** Assessment of patient’s opioid medication history/ current regimen, current prescribers/ dispensing pharmacies, home supply, identification of patients taking more than 60 mg MME per day.  **Preoperative medicine reconciliation review:** Telephonic comprehensive medication reconciliation review, pain assessment  **Inpatient postoperative treatment plans:** Standardized (opioid-naïve) or personalized (opioid tolerant) multimodal postoperative pain order set, pain monitoring  **Discharge treatment planning:** Preparation of outpatient pain regimen/ tapering plan based on the patient’s 24-hour pre-discharge opioid utilization.  **Discharge counselling:** Counselling sessions on opioids (risks, disposal etc.) and on tapering plan  **Follow-up appointments:** Medication, pain, and discontinuation status evaluation, pain management modification. | Clinical pain management pharmacists, clinical pharmacists, and student pharmacists | NR |
| Joo et al.  2020 | **Tapering plan generation:** The patient’s 24-hour oral opioid consumption before discharge is used to determine the amount of opioids (including the tablet number) that should be prescribed at discharge, and to generate an individualized tapering schedule. | NR | NR |
| Kukushliev et al.  2022 | **Tapering protocol generation:** Based on the patient’s 24-hour oral opioid consumption before discharge an individualized tapering protocol is generated.  **Tapering plan content:** The tapering protocol instructs the patient on the opioid dose and the frequency for each tapering day. | NR | NR |
| Tamboli et al.  2020 | **Tapering plan generation:** The patient’s 24-hour oral opioid consumption before discharge is used to determine the amount of opioids (including the tablet number) that should be prescribed at discharge, and to generate an individualized tapering schedule. | - Physician, nurse practitioner, or physician assistant from orthopedic surgery *(discharge opioid prescription)*  - Hospital pharmacist *(reviewing tapering plan)* | NR |

**MME:** Morphine milligram equivalents **NR:** Not reported. **NRS:** Numeric rating scale **PDMP:** Prescription drug monitoring program. **SMART:** Specific, measurable, attainable, relevant, time based. **SOWS:** Short opiate withdrawal scale
